# miRNA-338-3p/CDK4 signaling pathway suppressed hepatic stellate cell activation and proliferation

**DOI:** 10.1186/s12876-017-0571-3

**Published:** 2017-01-17

**Authors:** Bensong Duan, Jiangfeng Hu, Tongyangzi Zhang, Xu Luo, Yi Zhou, Shun Liu, Liang Zhu, Cheng Wu, Wenxiang Liu, Chao Chen, Hengjun Gao

**Affiliations:** 1 Department of Gastroenterology, Tongji Hospital, Tongji University School of Medicine, Shanghai, China; 2 Department of Respiration, Tongji Hospital, Tongji University School of Medicine, Shanghai, China; 3 Department of Gastroenterology, Changzheng Hospital, Second Military Medical University, Shanghai, China; 4 Department of Epidemiology, School of Public Health, Guangxi Medical University, Nanning, Guangxi China; 5 Digestive Endoscopic Center, Department of Gastroenterology, South Building General Hospital of PLA, Beijing, China; 6 Department of Gastroenterology, First Affiliated Hospital of Chinese PLA General Hospital, Beijing, China; 7 National Engineering Center for Biochip at Shanghai, Shanghai, China; 8 Department of Gastroenterology, Institute of Digestive Diseases, Tongji University School of Medicine, Shanghai, China

**Keywords:** Liver fibrosis, miR-338, *CDK4*

## Abstract

**Background:**

Activated hepatic stellate cell (HSC) is the main fibrogenic cell type in the injured liver. miRNA plays an important role in activation and proliferation of HSC.

**Methods:**

Our previous study examined the expression profiles of microRNAs in quiescent and activated HSC. Real-time PCR and western blot were used to detect the expression of Collagen type I (*Col 1*) and Alpha-Smooth Muscle Actin (*α-SMA*). CCK-8 and Edu assay was used to measure the proliferation rate of HSC. Luciferase reporter gene assay was used to tested the binding between miR-338-3p and Cyclin-dependent kinase 4 (*CDK4*).

**Results:**

We found overexpression of miR-338-3p could inhibit Col 1 and α-SMA, two major HSC activation markers, whereas miR-338-3p inhibitor could promote them. Besides, miR-338-3p overexpression could suppress the growth rate of HSC. Further, we found that *CDK4*, a pleiotropic signaling protein, was a direct target gene of miR-338-3p. Moreover, we found that overexpression of *CDK4* could block the effects of miR-338-3p.

**Conclusions:**

We found miR-338-3p is an anti-fibrotic miRNA which inhibits cell activation and proliferation. Our findings suggest that miR-338-3p/*CDK4* signaling pathway participates in the regulation of HSC activation and growth and may act as a novel target for further anti-fibrotic therapy.

## Background

Liver fibrosis is a common consequence of most chronic liver diseases [1]. Liver fibrosis received more attention until the hepatic stellate cell (HSC) was identified as the main ECM-producing cells in the injured liver [2]. Under the normal physiologic condition, HSCs reside in the space of disse and store a large amount of vitamin A. After suffering from liver injury, HSCs will be activated, and then proliferate, eventually transdifferentiate into myofibroblast-like cells [3].

microRNAs (miRNAs), short (~22 nt) conceding RNA molecules, can directly regulate gene expression by binding to the 3’UTR region of target mRNA to participate in lots of regulation of physiological process and diseases [4–8]. Recently, researchers focused on the role of miRNA in liver fibrosis pathophysiology to determine their regulatory effects on proliferation, differentiation of HSC [9–11]. Several abnormally expressed miRNAs were found and identified between quiescent and activated HSCs by using miRNA array or RT-PCR [12–19]. Previous studies have reported that miRNAs were critically involved in the activation of HSCs. Among them, miR-29b precursor allowed activated HSCs to switch to a more quiescent state [10]. Similarly, overexpression of miR-27a/b could lead HSCs to a quiescent phenotype [20]. microRNA-338 (miR-338), a newly identified miRNA, played a crucial role in a variety of carcinomas. Aberrant expression of miR-338 was closely related to cell proliferation, invasion, early detection and clinic pathologic variables in liver cancer, colorectal cancer, gastric cancer and neuroblastoma [21–25]. In previous research, our miRNA microarray data have found altered expression of miR-338-3p during culture activation of HSC [14]. However, little is known about the role of miR-338-3p in liver fibrosis.

Cyclin-dependent kinase 4 (*CDK4*) is found to be involved in cell cycle regulation. Activation of cyclin D—*CDK4* promotes the cell cycle progression through G1/S transition [26]. Inhibition of *CDK4* shows promising efficacy on advanced breast cancer [27]. In liver tissue and hepatoma cells, *CDK4/6* inhibition is a potent mediator of cytostasis [28]. However, whether the CDK4 participates in the fibrogenic process and regulates HSC activation and proliferation remains largely unknown. In this study, RT-PCR data suggested that miR-338-3p expression in fully activated HSCs were significantly decreased compared with that in quiescent HSCs. Transforming growth factor (TGF-β) is deemed to be the most potent fibrogenic cytokine. The results showed that there was a negative relationship between TGF-β and miR-338-3p. Therefore, we speculated that miR-338-3p was closely associated with HSCs function. Then, we found overexpression of miR-338-3p could suppress HSCs activation and proliferation while inhibition of miR-338-3p could promote HSCs activation and proliferation. Based on the Bioinformatics prediction, we found that *CDK4* was a potential target gene of miR-338-3p. Further luciferase reporter assay and RT-PCR confirmed their complementary binding. Moreover, our results indicated that overexpression of *CDK4* could partially block miR-338-3p-inhibited cell activation and proliferation.

## Methods

### Primary rat HSCs, cell lines and culture

The isolation method of primary rat HSCs was according to the previous literature [29]. Primary Rat HSCs, HSC-T6 and HEK293T (human embryonic kidney cell line) were kindly gifted from Dr. Gao (Tongji University, Shanghai, China). The primary cells and cell lines were cultured in DMEM (Dulbecco’s modified Eagle’s medium, Thermo, Waltham, MA, USA) containing 10% FBS (Fetal bovine serum, FBS, Gibco, Grand Island, NY, USA) at 37 °C in a humidified atmosphere of 5% CO2.

### Plasmid construction

Wild-type 3’UTR containing predicted miR-338-3p binding sites were amplified from HSC-T6 genomic DNA and inserted into the PGL3 luciferase reporter vector. Mutant 3’UTR was generated using the Quick Change Lighting Site-Directed Mutagenesis Kit (Agilent Technologies, Santa Clara, CA, USA). The *CDK4* expression vector was obtained by cloning the *CDK4*-coding sequence into the pcDNA.

### RT-PCR analysis

Total RNA was extracted from cultured HSCs using Trizol Reagent (Takara, Dalian, China). The primer sequences used for mRNA detection in this study were listed as follows: GAPDH (PF: CAGTGCCAGCCTCGTCTCAT, PR: AGGGGCCATCCACAGTCTTC); ColI (PF: ATCCTGCCGATGTCGCTAT, PR: CCACAAGCGTGCTGTAGGT); α-SMA (PF: CCGAGATCTCACCGACTACC, PR: TCCAGAGCGACATAGCACAG); *CDK4* (PF: GAAGAAGAAGCGGAGGAAGAGG, PR: TTAGGTTAGTGCGGGAATGAAT).

### CCK-8 assay and Edu assay

Cell proliferation was performed using CCK-8 assay (Dojindo, Japan) and Edu (Ribibio, Guangzhou, China) assay. For CCK-8, HSC-T6 was transfected with the miR-338 precursor, miR-338 inhibitor (Ribibio, Guangzhou, China) or pcDNA-*CDK4* in 96 well culture plates. Proliferation rates were tested at 24, 48 and 72 h after transfection. The EdU assay was conducted according to the protocol of Ribibio Edu Kit.

### Luciferase assay

Luciferase assay was performed with the Dual Luciferase Reporter Assay System (Promega, Madison, WI). Transfection was carried out in 48 well plates using Fugen (Roche). There were two groups. One was co-transfected with 200 ng wild-type-*CDK4-*3’UTR, 20 nm miR-338 precursor, and 20 ng Renilla. Another was co-transfected with 200mutant *CDK4* 3’UTR without binding site of miR-338-3p, 20 nm miR-338 precursor, and 20 ng Renilla. 48 h later, Firefly and Renilla luciferase activities were tested.

### Western blotting

Cells were lysed in SDS sample buffer. Antibodies against GAPDH (Biogot, 1:5000 dilution, Nanjing, China), Col1 (Abcam, 1:1500 dilution, Cambridge, MA, USA), α-sma (Sigma, 1:1000 dilution, Shanghai, China) and *CDK4* (Biogot, 1:3000 dilution, Nanjing, China) were used in this study. Signals were visualized with ImageQuant LAS 4000 (GE Healthcare Life Sciences).

### Statistical analysis

The statistical analysis in our study was performed by using SPSS 22.0. Data were given as mean ± SEM. Two tailed *t*-test was used to determine between two groups. Statistical significance level was set at *p* < 0.05.

## Results

### miR-338-3p was downregulated in fully activated HSCs

**Fig. 1:**
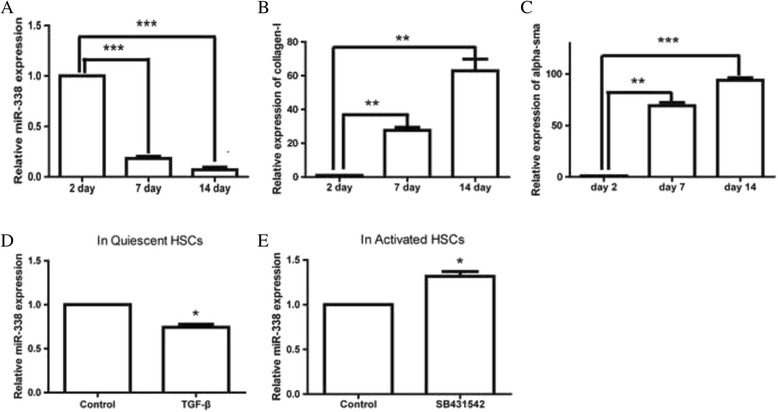
Expression of miR-338-3p is reduced in HSCs during culture activation. **a** miR-338-3p expression in the HSCs during culture activation. Data shown are means ± SD (*n* = 3), ***P < 0.001. **b** mRNA level of Col1 in the HSCs during cell culture. Data shown are means ± SD (*n* = 3), **P < 0.01. **c** mRNA level of α-sma in the HSCs during cell culture. Data shown are means ± SD (*n* = 3), **P < 0.01, ***P < 0.001. **d** miR-338-3p expression of quiescent HSCs was reduced upon TGF-β treatment. Data shown are means ± SD (*n* = 3), *P < 0.05. **e** miR-338-3p expression of activated HSCs was increased upon SB431542 treatment. Data shown are means ± SD (*n* = 3), *P < 0.05

Based on the microarray data, multitude ectopic miRNAs were detected in the HSCs. In our previous study, we isolated rat primary HSCs and extracted total RNA to perform miRNA microarray assay. Our attention was focused on miR-338-3p, a new underlying member of liver fibrosis. The microarray data showed that the expression of miR-338-3p was sharply reduced by 90% at day 7 (partially activated HSCs) [14]. To validate this finding, RT-PCR was carried out to measure the endogenous miR-338-3p expression in a quiescent state and an activated state. As primary HSCs were gradually activated during culture, we assessed miR-338-3p expression at day 2, day 7 and day 14 after isolation. Our data indicated that endogenous miR-338-3p expression was obviously reduced at day 7 and day 14 (Fig. 1a). Meanwhile, collagen type I (Col1) and α-sma (α-smooth muscle actin, α-SMA), two key biomarkers of HSCs activation, was gradually increased (Fig. 1b, c). In addition, we found that treatment with transforming growth factor (TGF-β, 2 ng/ml) in quiescent HSCs (day 2), the expression of miR-338-3p was reduced rapidly (Fig. 1d). When cells were fully activated (day 14), we treated them with SB431542, a potent and specific inhibitor of TGF-β and detected the level of miR-338-3p. The results suggested that the expression of miR-338-3p was increased compared to that in control group (Fig. 1e).

### Overexpression of miR-338-3p suppressed HSC activation while inhibition of it promoted HSC activation

**Fig. 2:**
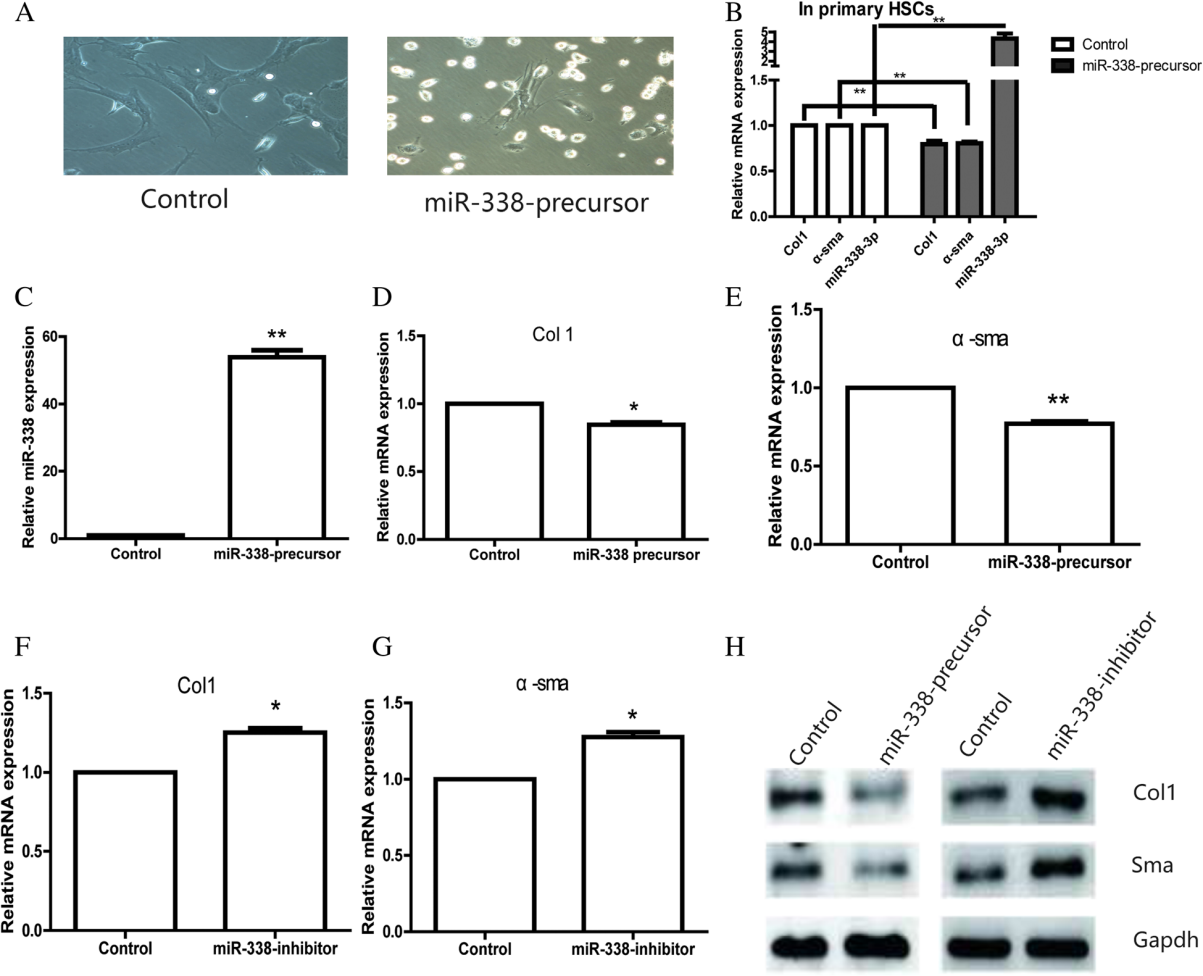
Overexpression of miR-338-3p could inhibit cell activation, whereas inhibition of miR-338 could promote cell activation. **a** The cell morphology of HSCs after transfecting with miR-338 precursor or negative control. **b** The expression of miR-338, Col1, and α-SMA in primary HSCs was tested after miR-338-precursor transfection. **c** miR-338-3p transfection efficiency was confirmed by qRT-PCR. Data shown are means ± SD (*n* = 3), **P < 0.01. **d** mRNA level of Col1 in the HSCs transfected with miR-338 precursor or negative control. Data shown are means ± SD (*n* = 3), *P < 0.05. **e** mRNA level of α-sma in the HSCs transfected with miR-338 precursor or negative control. Data shown are means ± SD (*n* = 3), **P < 0.01. **f** mRNA level of Col1 in the HSCs transfected with miR-338-3p inhibitor or negative control. Data shown are means ± SD (*n* = 3), *P < 0.05. **g** mRNA level of α-sma in the HSCs transfected with miR-338-3p inhibitor or negative control. Data shown are means ± SD (*n* = 3), *P < 0.05. **h** Protein level of Col1 and sma by western blot

As we discovered, miR-338-3p was significantly decreased during the process of activation, we transfected miR-338 precursor to repair its loss at the early stage of primary HSCs. On day 7, cells transfected with miR-338 precursor showed a more original shape with less peripheral protrusions. However, the control group cells showed a more irregular shape with more peripheral protrusions (Fig. 2a). As results showed in Figs. 1b, c and 2a, it seemed that there was a negative relationship between miR-338 expression and HSCs activation. We assumed that the expression of miR-338-3p was involved in this activation process. To confirm our hypothesis, we alter the miR-338-3p expression in primary HSC by transfecting miR-338 precursor. Interestingly, the expression of Col 1 and α-sma was slightly decreased due to the upregulation of miR-338-3p (Fig. 2b). Due to the low efficiency of transfection in primary cells, HSC-T6 cell line was further used to conduct the following studies. To replicate the results, HSC-T6 cell line was transfected with miR-338 precursor or negative control. 48 h later, cells were collected and transfection efficiency was confirmed by RT-PCR (Fig. 2c). Then the expression of Col1 and α-sma in two groups was measured using RT-PCR. As expected, the data showed miR-338-3p inhibited HSCs activation. The expression of Col1 and α-sma was respectively reduced as a result of miR-338-3p overexpression (Fig. 2d, e). Furthermore, inhibition of miR-338-3p could upregulate Col1 and α-sma which confirm their association on the other direction (Fig. 2f, g). Their expression was also confirmed at the protein level (Fig. 2h).

### Overexpression of miR-338 suppressed HSC-T6 proliferation

**Fig. 3:**
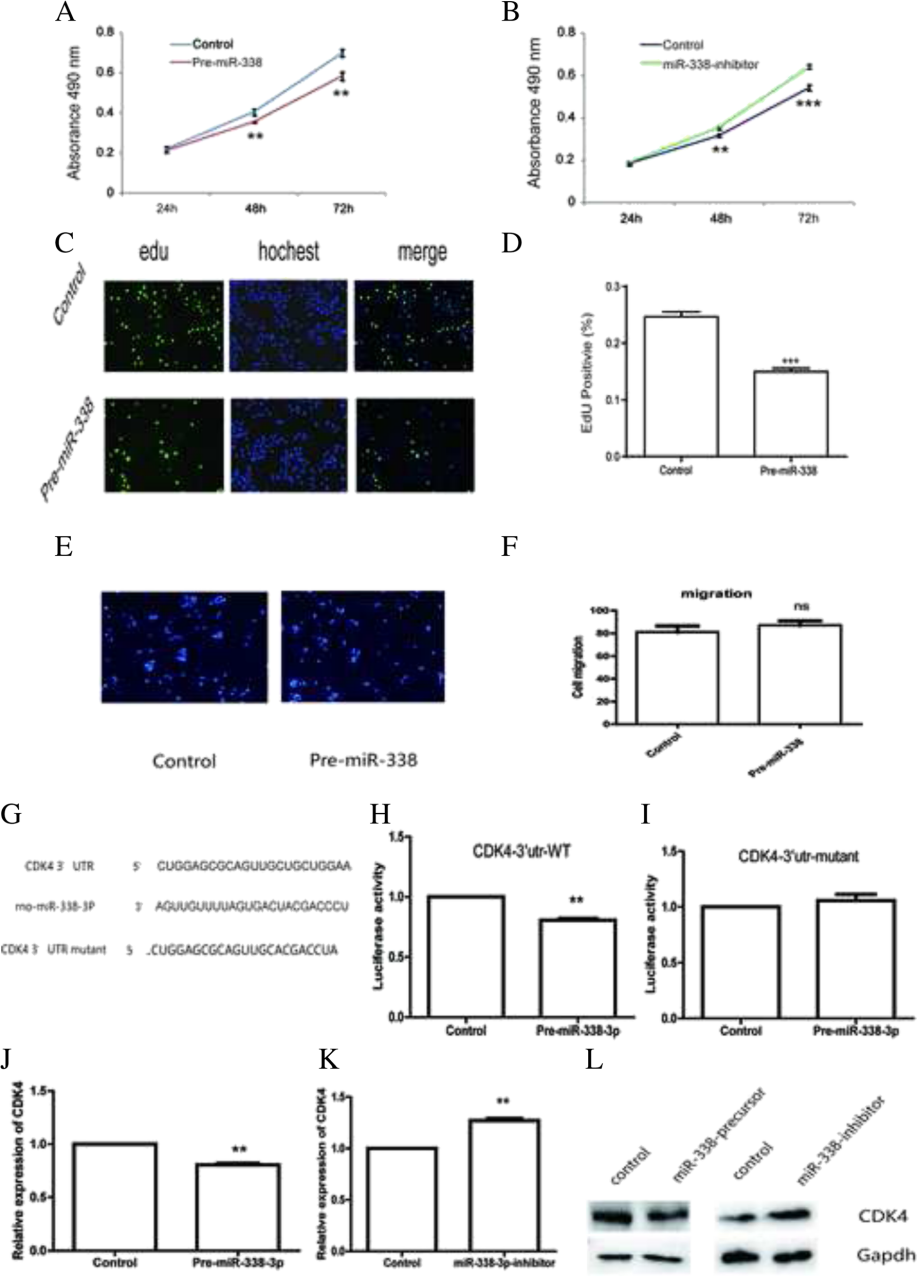
miR-338-3p regulates cell proliferation and CDK4. **a** The proliferation analysis of HSC-T6 transfected with miR-338 precursor or negative control. Data shown are means ± SD (*n* = 3). **P < 0.01 versus the corresponding control. **b** The proliferation analysis of HSC-T6 transfected with miR-338 inhibitor or negative control. Data shown are means ± SD (*n* = 3). **P < 0.01 versus the corresponding control. **c** Micrograph of HSC-T6 transfected with miR-338 precursor. **d** Edu incorporation assay demonstrated that miR-338 precursor reduced the proliferation of HSC-T6. Data shown are means ± SD (*n* = 3). ***P < 0.001. **e**, **f** HSCs migration was measured by transwell assay. **g** The predicted sequence of binding region between miR-338-3p and *CDK4*. **h** Luciferase activity of *CDK4* 3’UTR WT reporter vector co-transfected with miR-338-3p. Data shown are means ± SD (n = 3). **P < 0.01. **i** Luciferase activity of *CDK4* 3’UTR mutant reporter vector co-transfected with miR-338-3p. Data shown are means ± SD (*n* = 3). **j** mRNA level of *CDK4* in the HSCs transfected with Pre-miR-338-3p or negative control. Data shown are means ± SD (*n* = 3). **P < 0.01. **k** mRNA level of *CDK4* in the HSCs transfected with miR-338-inhibitor or negative control. Data shown are means ± SD (*n* = 3). **P < 0.01. **l** The expression of *CDK4* was restrained by miR-338-precursor, whereas recovered by miR-338 inhibitor

Next, we examined the impacts of miR-338 on HSCs proliferation. HSC-T6 were transfected with miR-338 precursor, inhibitor or corresponding negative control. In the CCK-8 assay, the cell growth curves suggested that overexpression of miR-338 significantly restrained HSC-T6 proliferation in a time-dependent manner (Fig. 3a.). Moreover, cells transfected with miR-338 inhibitor showed a higher proliferative ability (Fig. 3b). Edu incorporation assay also demonstrated that miR-338 precursor reduced the proliferation of HSC-T6 (Fig. 3c-d). Besides proliferation, we also assessed the role of miR-338-3p in HSCs migration. As Fig. 3e, f showed, miR-338-3p has no effects on cell migration.

### miR-338 repressed *CDK4* expression by directly binding to its 3’UTR region

**Fig. 4:**
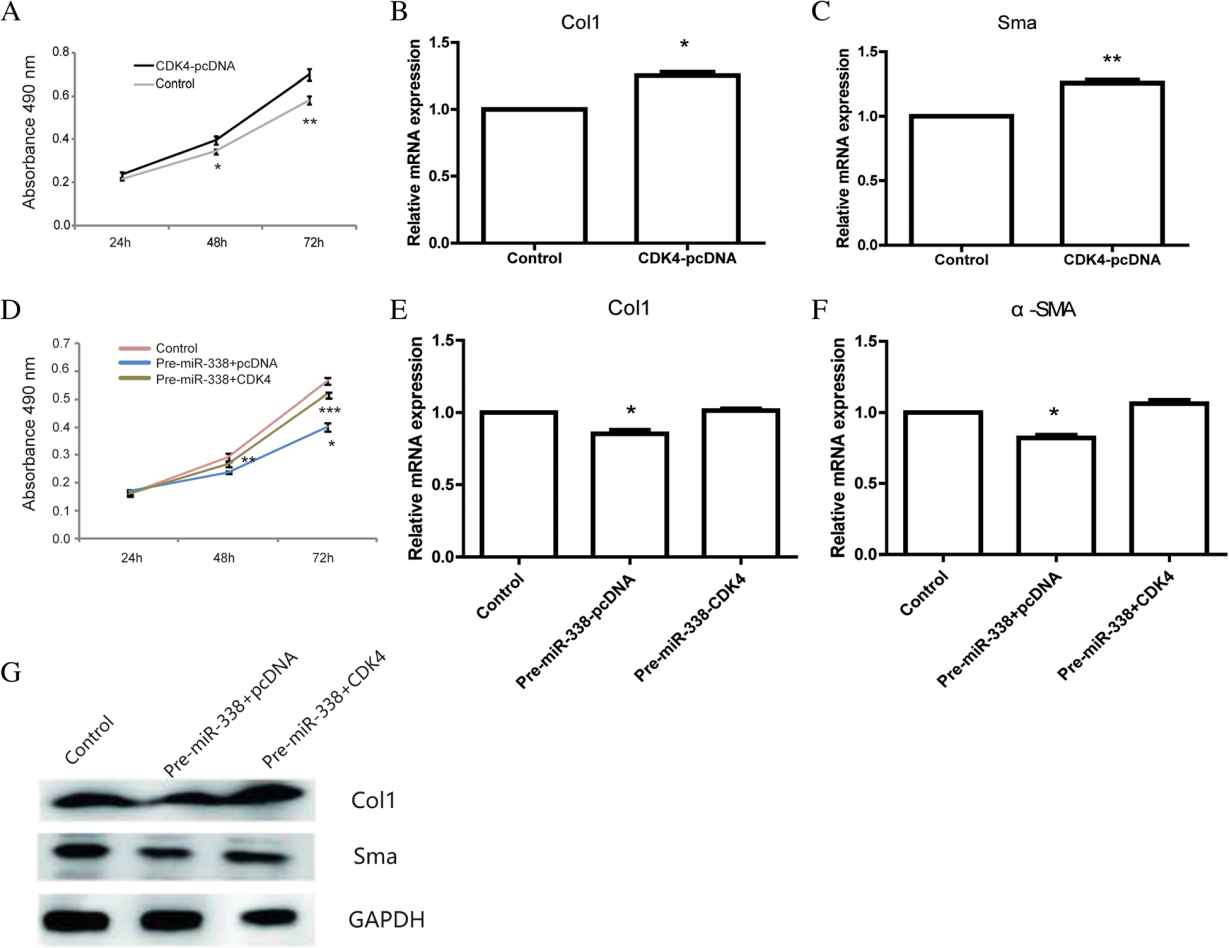
Overexpression of CDK4 could partially rescue the effects of miR-338-3p upon HSCs. **a** The proliferation analysis of HSC-T6 cells transfected with *CDK4* vector or empty control. Data shown are means ± SD (*n* = 3). *P < 0.05, **P < 0.01 versus the corresponding control. **b** mRNA level of Col1 in the HSCs transfected with *CDK4* vector or empty control. Data shown are means ± SD (*n* = 3), *P < 0.05. **c** mRNA level of α-sma in the HSCs transfected with *CDK4* vector or empty control. Data shown are means ± SD (*n* = 3), **P < 0.01. **d** The proliferation analysis of HSC-T6 cells co-transfected with miR-338 precursor and *CDK4* plasmid. Data shown are means ± SD (*n* = 3). *P < 0.05, **P < 0.01, ***P < 0.001. **e** mRNA level of Col1 in the HSC-T6 cells co-transfected with miR-338 precursor and *CDK4* plasmid. Data shown are means ± SD (*n* = 3), *P < 0.05. **f** mRNA level of α-sma in the HSC-T6 cells co-transfected with miR-338 precursor and *CDK4* plasmid. Data shown are means ± SD (*n* = 3), **P < 0.01. **g** Protein level of Col1, α-sma in the HSC-T6 cells co-transfected with miR-338 precursor and *CDK4* plasmid

Based on the prediction of Bioinformatics (TargetScan, PicTar), some genes are predicted to be the targets of miR-338-3p. Among them, we hypothesized *CDK4*, an oncogene in liver cancer, might be a putative target gene of miR-338-3p in liver fibrosis. The predicted sequence of interaction was showed in Fig. 3g. To test this prediction, 3’UTR with miR-338-3p binding sites were cloned into the PGL3 luciferase reporter vector. A mutant 3’UTR of *CDK4* with anti-sense mutation in the predicted sites was also constructed. The reporter construct was co-transfected into HEK293T with Renilla plasmid and miR-338 precursor or negative control. The wild-type *CDK4*3’UTR luciferase activity was suppressed due to miR-338-3p overexpression. By contrast, the activity of mutant-3’UTR-*CDK4* remained relatively unaffected (Fig. 3h, i). Additionally, *CDK4* expression was decreased in the miR-338 precursor group while increased in the miR-338 inhibitor group (Fig. 3j, k). miR-338-3p could also regulate *CDK4* at protein level (Fig. 4l). Taken together, these data strongly suggested that miR-338-3p repressed *CDK4* expression by directly binding to its 3’UTR region.

### *CDK4* rescued miR-338-inhibition of activation and proliferation of HSCs

Since miR-338 regulated cell growth, cell migration and cell invasion in liver cancer and colorectal carcinoma by targeting *CDK4* [21, 30], the role of *CDK4* in liver fibrosis remained unclear.

We conduct CCK-8 assay to determine the proliferation of HSC-T6 transfected with *CDK4* plasmid or control empty vector. The results showed that cell transfected with *CDK4* plasmid displayed a higher proliferative capacity compared with the control group (Fig. 4a). Besides, we found cells transfected with *CDK4* could promote cell activation with upregulation of Col1 and α-Sma (Fig. 4b, c). We co-transfected HSC-T6 cells with miR-338 precursor and *CDK4* plasmid to investigate whether *CDK4* would rescue the inhibition effect of miR-338 on the cell activation and proliferation or not. The results suggested that overexpression of *CDK4* partially block the repression effect of miR-338 on the activation and proliferation of HSC-T6. The growth curves of three groups (Control, Pre-miR-338/pcDNA, Pre-miR-338/CDK4) were shown in Fig. 4d. The expression of activation associated markers, Col1 andα-Sma, was showed in Fig. 4e-f.

## Discussion

Liver fibrosis is a scarring response to liver damage. It’s a common pathological process for most of the liver disorder. A small number of patients go on to progress cirrhosis and/or hepatocellular carcinoma. Fortunately, liver fibrosis can be reversed if the inflammation was controlled [31].

Aberrant expression of miRNAs have been involved in liver fibrosis and regarded as a potential treatment strategy. Intervening miRNAs expression could assist activated HSCs to return to a quiescent phenotype. miRNA microarray and RT-PCR was carried out to determine abnormally expressed miRNAs during HSCs activation. Our results suggested that miR-338-3p was significantly downregulated in this process. miR-338 is located on chromosome 17q25.3 with a length of 22 nt and produces two mature forms, miR-338-3p and miR-338-5p. miR-338 was first reported in neurodegeneration and gradually studied in various disease [32]. In hepatocellular carcinoma, miR-338 downregulation was associated with tumor size, TNM stage, vascular invasion and in trahepatic metastasis [21, 22]. In colorectal carcinoma, miR-338 expression was significantly increased in both blood and tissue samples. It might appear to be a potential biomarker for early detection in colorectal carcinoma [23]. In gastric carcinoma, miR-338 was epigenetically silenced and its reduction was related to pathological variables. Overexpression of miR-338 could suppress cell proliferation, migration, invasion and tumorigenicity [24]. Moreover, combined with other six miRNAs, miR-338 could be used to predict gastric cancer prognosis [33]. Despite in cancer, miR-338 was also involved in idiopathic pulmonary fibrosis [34].

This is the first study to identify the biological function of miR-338-3p in liver fibrosis. Our results demonstrated that miR-338 precursor transfection suppressed the activation and proliferation of HSC-T6, whereas inhibition of miR-338-3p promoted cell activation and proliferation.

To understand the underlying mechanism of miR-338-mediated inhibition of proliferation, we identified *CDK4*, a member of the cyclin-dependent kinase family*,* as a candidate target gene. *CDK4* usually work with Cyclin D to regulate the cell cycle in G1/S stage. Aberrant activation of *CDK4* was closely associated with various kinds of carcinomas. *CDK4* expression is significantly upregulated in lung cancer tissues and function as an important element for cell proliferation [35, 36]. In breast cancer, inhibition of *CDK4* can induce G1 arrest [37]. These observations suggest that inhibition of *CDK4* might be beneficial for cancer treatment. An increasing body of clinical trials targeting *CDK4* has been launched. However, the role of *CDK4* in liver fibrosis remains largely unknown. In this study, Luciferase reporter assay showed that there was a combination of miR-338 and *CDK4*. Hence, we deduced that miR-338-3p inhibited HSCs’ activation and proliferation likely through silencing CDK4. Our data indicated that restoring CDK4 expression could partially rescue miR-338-inhibited cell activation and proliferation.

## Conclusions

In conclusion, our study identified a new anti-fibrosis miRNA that may play an important role in the development of liver fibrosis.

## ᅟ

CDK4: Cyclin-dependent kinase 4
Col 1: Collagen type I
HSC: Hepatic stellate cell
miR-338: microRNA-338
α-SMA: Alpha-Smooth Muscle Actin
